# An Integrated Success Model of Internet of Things (IoT)-Based Services in Facilities Management for Public Sector

**DOI:** 10.3390/s22093207

**Published:** 2022-04-22

**Authors:** Norliza Sidek, Nor’ashikin Ali, Gamal Alkawsi

**Affiliations:** 1 College of Graduate Studies, Universiti Tenaga Nasional, Kajang 43000, Malaysia; pt20718@utn.edu.my; 2 Institute of Sustainable Energy (ISE), Universiti Tenaga Nasional, Kajang 43000, Malaysia; gamal.abdulnaser@uniten.edu.my

**Keywords:** Internet of Things, facility management, public sectors, technology readiness, quantitative method

## Abstract

The rapid growth of the Internet of Things (IoT) has vigorously affected government by enhancing quality and efficiency of public services. However, the application of IoT-based services in public sectors is slow, despite its benefits to citizens. Research is needed to deepen understanding of the factors that influence the successful implementation of facilities management as the Internet-of-Things-based services in public sectors. An integrated model is developed and validated to extend the DeLone and McLean IS success model by including technology readiness and other identified factors which impact the use of facilities management of IoT-based services in public sectors from the perspective of employees. An online questionnaire was developed and distributed to employees from all local authorities throughout Malaysia, and 187 usable responses were collected. The partial least squares structural equation modelling approach was used to test the model, with 90.8% of the variance in IoT-based services, suggesting an acceptable model fit with seven out of nine hypotheses were supported. Thus, the empirical evidence exerts significant effects of technology readiness towards the success of IoT-based facility management in the public sector.

## 1. Introduction

The emerging technologies of Internet of Things (IoT) have brought dramatic changes to the public sector in improving the quality of their public services. The application of IoT to facilities management (IoT-FM) is a government initiative for improving public service delivery, and has been identified as a crucial domain in smart cities [1,2,3] Traditionally in facilities management (FM), particularly in building management, the activities of collecting, processing and modelling ‘as-is’ information for existing buildings is time-consuming and costly. The emergence of IoT-based services (IoTbs) has changed the traditional approaches of managing public facilities and opened new perspectives for intelligent FM that deliver the effective management of resources [4]. IoT-based services in FM (IoTbs FM) involve the application of critical decision-making, which takes into consideration the environmental impact, safety issues, and challenging situations within the facilities management of buildings. These considerations extend to the management of road maintenance, parks and playgrounds, garbage disposal, and landscaping, which requires cross-functional coordination and commitment by employees to meet the specific purposes.

IoTbs in FM functions by collecting real-time information for facilitation, tracking the location of users and objects, processing and analysing the conditions of facilities, and suggesting a specific solution, along with predicting the risk status of facilities. Employees can obtain real-time information on the condition of facilities transmitted wirelessly to their devices (e.g., smartphones, personal computers, and laptops) to improve work performance, enhance the management of public facilities and increase the quality of life among the citizens. Additionally, IoTbs in FM has capabilities to support real-time alert notifications and two-way communication between employees and citizens. Hence, the employees of local authorities can continuously monitor the condition of the facilities remotely over a cloud server.

Traditional management practices mainly involve the planned preventive maintenance performed based on a timed schedule to detect faults before they occur, and corrective maintenance is carried out afterwards. IoTbs make predictive and condition-based maintenance possible to reduce the waste of resources and cost, as well as limit downtime. IoT in FM has been adopted by many countries, such as Norway, whereby data obtained by IoT devices may support a holistic view of a building condition, which can subsequently enhance FM development. IoTbs for FM development in Hong Kong has improved traditional construction methods by removing cast-in-situs small prefabrication factories that benefit from mass production [5]. This new FM approach is also considered as a feasible and economical way to construct “as-is” building information modelling (BIM) (‘as-is’ BIMs), and the length of time required for the (‘as-is’ BIM) can be reduced by using IoT technologies (e.g., sensors, cameras, and smartphone). Another example is related to the operation and maintenance services of FM.

Although IoT in FM may bring innovation to public sectors, this paradigm shift towards technological change may not have reached its potential. Many initiatives are still at emerging stages, which results in a gap between expected outcomes and achieved results. Related reports have argued that although more than two-thirds (70%) of public agencies are evaluating the potential of emerging technologies, only a small percentage (25%) could progress beyond the pilot phase to full implementation [6]. Thus, government initiatives in digital transformation are increasingly being questioned, with most outcomes fell short of expectations [7]. Thus, the rate of failure for government information technology (IT) projects is argued to be abnormally high in many countries [8] One of them, the United Kingdom (U.K.), have government agencies wasting USD 4 billion on failed IT projects, achieving only a 30% success rate [9]. Resistance to technology and a lack of usage of information systems have long been recognized as causes for project failure [10,11]. 

Previous studies have shown that many organisations fail to achieve the potential benefits of IoT and earn the returns on investments due to underutilisation by employees [12,13]. Employees must first use the IoT to create value for the public [14] According to the annual Society for Information Management (SIM) survey, change management and the accompanying resistance issues are among the top ten challenges facing Chief Information Officers (CIOs) [15]. New technologies can affect changes in the work routines; thus, employees may be reluctant to accept and use IoTbs. 

Empirical evidence suggests that the implementation of emerging technologies does not guarantee the effectiveness of services available, unless the concerns and abilities of employees in using the systems are addressed [16]. Additionally, most of these studies have argued that public sectors which have adopted IoTbs face internal resistance from employees [17,18,19,20]. Many studies focus on the value creation of IoT to citizens [21,22]; however, the role of employees in accepting and using IoTbs to deliver public services should not be overlooked, particularly in FM. IoTbs in FM are the backbone of smart cities [21,23] and smart cities are the backbone of smart government [24,25]; therefore, investigation on the use behaviour among IoTbs in FM employees is crucial for implementing successful smart services in the public sector. 

Technology readiness is described from the perspective of individual traits [26] (Parasuraman, 2000), and has been identified as a barrier towards the implementation of smart initiatives in public sectors.According to empirical evidence from the existing literature, smart initiatives in organizations have failed because employees resist when they are not ready to change and use IoTbs to increase their performance [27,28]. However, little attention has been given towards the impact of technology readiness towards the success of IoTbs. Employers and designers of IoTbs should be aware that by tackling technical issues, such as information quality, system quality, and support services in IoTbs, the desired objective of implementation may not necessarily be achieved as the needs of the employees are not addressed. This argument has been cited in numerous past studies, whereby although technical factors were addressed, human factors associated with individual traits need to be given sufficient attention. In other studies, peer influence plays a vital role in promoting employees on using new technology [29,30]. 

This study aims to identify the factors that influence the behaviour of employees in using IoT-based services and to develop an integrated model that address technical and human factors which impact the use of IoT-based services in public sectors from the perspective of employees. This research contributes to the literature by discussing acceptance and success factors related to the behaviour of users, which in this study, were the employees. The addition of technology readiness constructs and subjective norms to the success model in the IoTbs of FM model contributes to extending the theoretical and empirical evidence on the ways technology readiness and the subjective norms of employees influence the success of IoTbs, which is not covered in prior research. From a practical point of view, the proposed model assists public sectors to be concerned on pertinent factors required for implementing IoTbs in general, and FM in particular. 

The remainder of this paper is organized as follows. Section 2 reviews the related work, followed by Section 3, which explains the theoretical concept and choices behind the model of this study. The research methodology is assessed in Section 4. The analysis findings are summarized in Section 5, followed by Section 6, where the results of the analysis are discussed. Section 7 presents the research contribution. The implications of the findings are outlined in Section 8; then, Section 9 shows the research limitations and suggests future works, and Section 10 concludes the article. 

## 2. Literature Review

### 2.1. IoT-Based Services in Facilities Management

IoT-based services in FM (IoTbs FM) involve the application of critical decision-making, which takes into consideration the environmental impact, safety issues, and challenging situations within the facilities management of buildings. These considerations extend to the management of road maintenance, parks, and playgrounds, garbage disposal, and landscaping, which requires cross-functional coordination, as well as commitment by the employees to meet the specific purposes. The emergence of IoT-based services (IoTbs) has changed the traditional approaches of managing public facilities and opened new perspectives for intelligent FM that delivers the effective management of resources [4]. For example, in Norway, data obtained by IoT devices may support a holistic view of a building condition, which can subsequently enhance FM development. On the other hand, IoTbs for FM development in Hong Kong has improved the traditional construction method by removing cast-in-situs of small prefabrication factories that benefit from mass production [5]. In Greece, an intelligent system was able to reduce the costs of infrastructure and improve road user safety [31].

IoTbs in FM function by collecting real-time information for facilitation, tracking the location of users and objects, processing and analysing the conditions of facilities, and suggesting a specific solution, along with predicting the risk status of facilities. Employees can obtain real-time information on the condition of facilities transmitted wirelessly to their devices (e.g., smartphones, personal computers, and laptops) to improve work performance, enhance the management of public facilities and increase the quality of life among the citizens. Additionally, IoTbs in FM have the capability to support real-time alert notification and two-way communication between employees and citizens. Hence, the employees of local authorities can continuously monitor the condition of the facilities remotely over a cloud server. For example, the corresponding authority within the garbage disposal sector would be informed through a real-time notification from the sensors placed in the dustbin when the dustbin is filled up and sends garbage trucks to the notified location to collect waste, which can optimise costs and save time. This technology is used in Bangladesh, whereby the garbage bins are equipped with sensors to detect and alert employees when they are full [32], which reduces the time needed for employees to check the garbage bins before being emptied manually. Employees can also use IoTbs to remain proactive in road maintenance. In France, IoT road sensors can provide real-time data from roadways to help reroute traffic away from dangerous places [33]. Thus, IoTbs can bring several benefits to FM in terms of providing real-time monitoring [34] or intelligent risk prediction [35], which further enhances the work and public service performance of employees.

Table 1 shows that FM has a broad scope of operation across industries, which does not solely focus on building management. The table demonstrates the scope and devices of IoTbs for FM, which later link the features in IoTbs for FM to the general characteristics of IoTbs. The scope applied in this research is similar to that proposed by [3], who suggested that IoTbs for FM should not only be limited to building management, but should also include a wide spectrum of public facilities such as animal control (e.g., stray dog control), landscaping services (e.g., removing fallen trees), road services (e.g., potholes, cracks) and waste management (e.g., garbage disposal services).

### 2.2. Previous Studies on IoT-Based Services Success

The literature on IoTbs success is divided into two perspectives: the research domain and countries. In terms of the research domain, some IoTbs have been already developed in healthcare [40], smart homes [41,42], manufacturing [43], farming [44], smart meters [45,46,47], education [48], smart cities [21] and the retail industry [49] However, this review found that research in the context of FM is limited. 

From another perspective, IoTbs have been implemented in many countries, including China [50], The Netherlands [51], Taiwan [52], Korea [53,54] India [21], the United States [55] Jordan [56] and Malaysia [57]. However, empirical studies of IoTbs in developing countries are still needed to achieve the Sustainable Development Goals (SDGs) of the United Nations. 

Another part of this review concentrated on the theories that have been used by IoT researchers. Several theories have been applied to evaluate the factors affecting IoTbs usage, and they differ from country to country, from context to context and from respondent to respondent. Previous studies have identified factors which influence the use of IoT from the perspective of acceptance models, and adopted the Technology Acceptance Model (TAM), Technology Readiness Index (TRI), Unified Theory of Acceptance and Use of Technology (UTAUT), Theory of Planned Behaviour (TPB) and Theory of Reasoned Action (TRA). This review evaluates how factors in the theories interact with each other to affect IoT utilisation, which then led this research to focus on the technical and human factors influencing the success of IoTbs in general, rather than on the perception or acceptance of specific IoT characteristics. As a result, theories from information systems (IS) and social science in general, and the D&M IS success model, TRI, and TAM in particular, were used as the underlying theoretical framework for this research. Although the TRI and D&M IS success model have been suggested as potentially useful in examining the acceptance and use of IoTbs, there are limited studies of IoT success that use the D&M IS success model, particularly its association with TRI. Furthermore, there is insufficient evidence on the impact of TR on IoT success. 

## 3. Theoretical Framework 

### 3.1. Justification for Selecting the D&M IS Success Model

The D&M IS success model brings multiple measures of success and is comprehensive in measuring IS success. It captures not only the technical qualities, but also human perceptions of users’ satisfaction and usefulness of the system. Previous studies have suggested that success and its measurement are different based on the characteristics of the system and the organisation; therefore, the model should be modified [58,59]. In the case of IoTbs that rely on the connectivity and smartness of objects as well as information provided by the application, the D&M IS success model offers successful measures for the quality of the system, information and service and measures for users’ satisfaction and usefulness in using IoT. In achieving the objectives of this research, the D&M IS success model is reformulated to combine different theoretical perspectives that may increase the values of the DeLone variables. Additional variables were incorporated from the literature to extend this model to reflect this perspective. The model was extended to include the variables of the Technology Readiness Index (TRI) and the subjective norm (SN) as variables that influence the success of IoTbs.

The D&M IS success model was used as a base model in this research. The model for this research was also developed by comparing it with the IoT success model [21], because it is the only IoT model validated in prior research that investigates the successful implementation of IoT in smart cities. According to [60], developing the current research model based on existing models may result in the creation of well-developed research streams because it establishes the construct’s nomological validity. The nomological validity requires the whole domain of the construct to be sufficiently captured in the measurement items, where the construct and their relationships to each other are tested in a broad variety of settings [61]. Hence, reusing existing research models will assist in the interpretation of results and significantly save time as opposed to creating entirely new models because the model has been used and the results can be compared [62].

### 3.2. IoT-Based Services Success Model for Facilities Management

**Figure 1:**
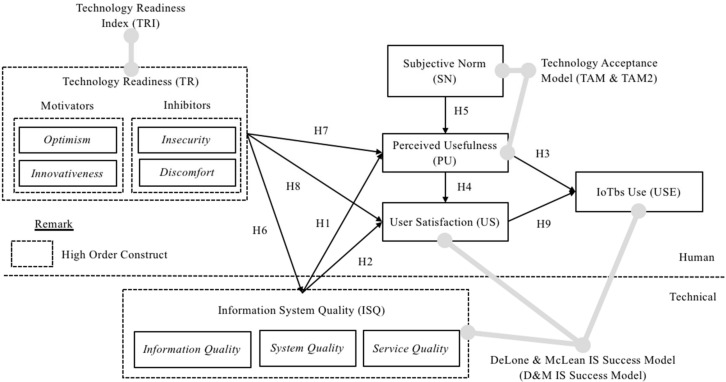
Research model.

Figure 1 shows the proposed model for the current study. The model was formulated to address the current research problem. A comparison between the current research model and [21] IoT success model was made. The proposed model was modified, refined, and tailored to fit the FM context as follows: (1) the actual usage of IoT was replaced with IoTbs Use; (2) perceived intention to use the IoT was replaced by perceived usefulness (PU); (3) new constructs, i.e., technology readiness (TR), and subjective norm (SN), were added to help explain variations in employees’ perception of IS quality (ISQ), usefulness, and user satisfaction (US); (4) the simultaneous causality between PU and US in the D&M success model was replaced with one-way causality; and (5) the perceived net benefit (NB) of IoT has been removed. 

The first difference was between the dependent variables. The actual usage of IoT was replaced with the IoTbs Use construct. System use was also noted as the actual usage of the system [63,64]. In [21] IoT success model, the actual usage of IoT was measured by using a subjective approach (self-reported data). The same approach was used in this research. Hence, it was considered that the replacement did not affect the outcome and functional characteristic of the construct. 

The second difference was the replacement of the perceived intention to use IoT with PU due to its reliability and validity as a predictor of IS usage, which was confirmed by [65]. Seddon and Kiew [66] proposed PU as a determinant of US and as a central success factor in the modified version of the D&M IS success model. Furthermore, Venkatesh and Morris [67] found that PU is an important factor influencing US towards the use of IS technologies. Reflecting on the relevance of PU as an essential factor in establishing employees’ satisfaction, and system use is affected through PU in situations where an IoTbs is not mandatory, PU was added to replace the intention to use IoT construct.

The third difference was the existence of additional factors, namely TR and SN. Both factors have been included in the research model as a determinant of IoT success in the FM context. Although many studies in the literature argued that these factors have significant influences on the adoption and use of IS, inconsistent evidence has led to further investigations. Hence, the SN and TR constructs were added to investigate the influence of the individual differences on ISQ, employees’ PU and US. 

The fourth difference was the simultaneous causality between PU and US, replaced with one-way causality (only from PU to US). Seddon and Kiew [66] suggested that PU causes US, not vice versa. Thus, the current research assumed that a higher PU level will lead to higher US with IoTbs. The fifth difference was the exclusion of the perceived NB of IoT. Wu and Wang [68] claimed that there is little consensus in the literature on how NB should be measured objectively. They highlighted that NB are usually determined by the perceptions of those who use the IS. Following the empirical studies of the KMS Success Model by Ali et al. [69] and Wu and Wang [68], this research interprets the NB as an IoTbs Use by arguing that IoTbs Use should be a behaviour that reflects the expectation of IoT benefits from using an IoTbs. Therefore, IoTbs Use should be a consequence of IoT success, rather than a determinant of IoT NB. 

Without ignoring the basic structure of the D&M IS success model, this research interpreted NB as system use and replaced the intention to use with PU, which is closely related to the TAM model. In TAM, perceived ease of use (PEOU) is conceptualised as an antecedent of PU, whereas PEOU and PU significantly influence user attitudes towards using technology. PEOU in TAM is somewhat related to IoT system quality (specifically refer to the ease-of-use characteristic for system quality (SQ) in the current research model). In TAM, PEOU is hypothesised to have direct impacts on intentions to use via PU. Meanwhile, in the present research model, ISQ was hypothesised to have positive effects on PU and indirect positive effects on IoTbs Use.

### 3.3. Outcome Variable of IoT-Based Services Success

It is logical that if IoTbs ceases to be in use, it cannot provide any benefit to employees; thus, it cannot be seen as successful. Therefore, IoTbs success, by any reasonable measure, is associated with IoTbs use. Although past studies have mainly examined the behavioural intention to use IoTbs, the examination of use behaviour on using IoTbs was less observed. Hence, this study used IoT-based Services Use (IoTbs Use) as the outcome variable in a model of IoTbs success, in which this variable indicates IoTbs success.

System use is one of the most frequently assessed variables in measuring IS success [70]. Similarly, in this research, IoTbs Use was interpreted as representing an IoT success. As Seddon [71] pointed out, when the use is not mandatory, the system use is a good proxy for IS success. Although IoTbs can be enforced as mandatory services among LAs’ employees, the conflict of regulatory power between federal, state, and local authorities (LAs) causes the use of IoTbs to depend on their free intentional will. As such, it can be stated that this research was conducted based on voluntary usage, and not mandatory usage, which is similar to the majority of prior studies of this domain.

### 3.4. Independent Variables of IoT-Based Services Success

The ISQ, PU, US, and IoTbs Use for IoTbs success are factors derived from the D&M IS success model, which was the underlying theory in this research. In addition, the TR was adapted from TRI by Parasuraman and Colby [72], and SN was derived from Technology Acceptance Model 2 (TAM2) by [73]. The TR and SN factors are integrated into the D&M IS success model to form the IoTbs success model and further explore the effect of human and technical factors on IoTbs utilisation. A series of hypotheses were developed from the earlier theoretical discussion and justified one by one, with an emphasis on their relevance in the IoTbs for FM context.

#### 3.4.1. Information System Quality

Due to the existence of a large number and heterogeneous devices, researchers are encouraged to improve IoT quality for guaranteed services [74]. Overall ISQ includes IQ, SQ, and SVQ, and has been studied as a high-order construct [75,76,77,78] The results unanimously supported the view that a positive relationship existed between ISQ, PU, and US. In this research, it is argued that high qualities of IS are critical in IoTbs because it affects employees’ work performance, which may have severe implications on citizens’ quality of life. It is posited that a high ISQ affects employees’ PU and their satisfaction with IoTbs which, in turn, influences the use of IoTbs to improve their job performance. Any glitches in IoTbs operation due to poor ISQ may potentially result in the working environment’s uncertainty, and will substantially impact the employee’s perception and satisfaction because employees are working under time pressure with inadequate resources. In view of the discussion above, the following hypotheses are justified:

**Hypothesis** **1** **(H1).** *Higher ISQ leads to higher PU.*

**Hypothesis** **2** **(H2).** *Higher ISQ leads to higher US.*

#### 3.4.2. Perceived Usefulness

Perceived usefulness (PU) has been used in studies relevant to TAM; however, the construct’s operationalisation differs according to the technology under study. PU is defined in the IoT context as individuals who perceive that utilising IoT can improve their satisfaction and working performance [79]. Mertens et al. [80] highlighted that people will only use a system if they find it useful. Prior IS research confirmed the effect of PU on system use [65,81]. Employees in the public sector care about their competence to contribute to their organisation’s success [82]. Therefore, if employees perceive IoTbs as useful in enhancing their work performance, they are more likely to use IoTbs functionalities. Furthermore, if IoTbs is perceived as useful, it is more likely that the system will satisfy the expectations of the employees, and thus employees are more likely to be satisfied with the IoTbs. In view of the discussion above, the following hypotheses are justified:

**Hypothesis** **3** **(H3).** *Higher PU leads to higher IoTbs Use.*

**Hypothesis** **4** **(H4).** *Higher PU leads to higher US.*

#### 3.4.3. Social Norms

Social pressures or social norms refers to the influence of colleagues, friends, or others who reinforce the intent to adopt new applications [81]. In IS studies, these aspects are usually assessed through SN constructs. In the context of IoT, Chang et al. [83] explored factors affecting the behavioural intention to adopt hearing aids in smart cities. They revealed that there is a significant relationship between SN and individuals’ intention to adopt. Within the same research context, Mital et al. [84] confirmed that SN had a significant direct positive effect on individuals’ intention to use smart devices in India. Therefore, further investigation is required, especially in the FM context. The TRI and D&M IS success model do not explicitly include any social variables; therefore, SN in this proposed research model may capture unique variance in attitudes and intentions. Based on the discussions above, it was suggested to include SN as a factor influencing the individual’s perception of usefulness towards IoTbs implementation’s success. Thus, the following hypothesis was developed:

**Hypothesis** **5** **(H5).** *Higher SN leads to higher PU of IoTbs.*

#### 3.4.4. Technology Readiness

The correlation between TR and their propensity to employ new technology is empirically confirmed by [85]. It is also a crucial determining factor in technology usage [86]. This research suggested that TR might influence the usage of IoTbs. Although previous research confirmed that TR has a significant direct effect on PU and US, TR’s role in ISQ is still under debate, particularly in the context of technology-based services and IoT. In relation to this, Roy et al. [87] claimed that the relationship between TR, PEOU, and PU is intuitive. The PEOU is somehow related to the ease-of-use characteristic of ISQ; thus, it is worth further investigating the role of TR as an antecedent of ISQ in the IoT context, with the assumption that higher levels of TR will increase employees’ views on ISQ of IoTbs. This research proposes that when employees use IoTbs, the TR (i.e., negative or positive feeling) will influence the overall quality of IoTbs.

Employees with high TR are less likely to focus on negative events and confront technology more openly. Moreover, they are more likely to think that they might miss certain benefits if they do not try out new technology. In their study regarding self-service technology, Van Huy et al. [88] determined the effect of TR on PU. The causal links between TR and usefulness perceptions were also confirmed by [89,90,91] in IoT studies. For instance, Roy et al. (2018) reported that people with higher TR are more likely to perceive smart technologies as more useful. Somsit et al. [92] found the same significant relationship between TR and PU in smart farming adoption. However, Kamble et al. [93] claimed that there was no significant relationship of TR with the user’s PU in blockchain technology adoption. Within this research context, it is argued that people with high levels of TR are more likely to form favourable impressions of the usefulness of IoTbs. 

Satisfaction is modelled as an outcome in the TR research of technology adoption [94], because TR may give a greater potential for good outcomes, leading to higher incentives for adopters. In the context of this study, it may be assumed that a higher degree of TR will result in employees receiving more benefits, and thus achieving better satisfaction from IoTbs.

In this study, TR was theorised to be an antecedent of ISQ, PU, and US, which subsequently affects employees’ use of IoTbs. Based on the discussion above, the following hypotheses were developed:

**Hypothesis** **6** **(H6).** *TR leads to higher ISQ.*

**Hypothesis** **7** **(H7).** *TR leads to* *higher* *PU.*

**Hypothesis** **8** **(H8).** *TR leads to higher US.*

#### 3.4.5. User Satisfaction 

US is defined as the degree to which individuals believe that the system is available to meet their requirements [95]. Low levels of US can reflect tension, frustration, and resentment, leading to inefficiencies in system usage [96]. Meanwhile, high levels of satisfaction can encourage better IS usage and influence employees’ work–life in the adoption of IoTbs for smart government [97]. US has been identified and empirically supported as a relevant indicator of IS success [98], and should be an essential component of IoTbs success evaluation. In the studies of emerging technology, for instance, IoTbs, which use wireless devices such as smartphones, it was found that US positively affects the use of mobile services [99]. In another study, in the field of smart cities in India, Chatterjee et al. [21] confirmed that the US contributed to the use of an IoT.

From the present research perspective, it is posited that the employees’ use of IoTbs is associated with their satisfaction in using the IoTbs. If employees perceive that the IoTbs do not fit with their needs, they will remain unsatisfied and reluctant to use the system. However, if the system meets employees’ needs, they will be satisfied and pleased to use the system. Consequently, the following hypothesis is justified:

**Hypothesis** **9** **(H9).** *Higher US leads to higher IoTbs Use.*

## 4. Research Methods

This research used a questionnaire as the main instrument of data collection. This section describes the development process of the survey instrument.

### 4.1. Survey Design

A pool of items from multiple prior studies were combined to ensure that the content coverage fitted the domain of IoTbs success in FM. The existing measurement items were adopted, with very few adjustments; only when adequate quality measures were available in the literature. They were reworded if necessary to fit the study’s context and to ensure that the appropriate content which was not captured was added in the adopted base measurement. 

This research applied the Likert scale format in the survey instrument. In this type of scale, a declarative statement is presented, followed by response options with varying degrees of agreement or endorsement of the statement [100]. A seven-point Likert scale was used for all measurement items. The instrument translation process followed the completion of the determination of scale. This research was performed in Malaysia and the existing instruments were written in the English language; therefore, the questionnaire had to undergo through a translation process using the back-translation method to determine if the translated items retained their original meanings. 

### 4.2. Content Validity 

Content validity was assessed to ensure that all the fundamental aspects of the particular constructs were reflected in the measurement items. Simultaneously, the content validity method was applied to acquire feedback on the translated instrument’s clarity and wording. Following the guidelines from Davis [101] and Rubio et al. [102], this research selected a group of experts from academia and industry, competent in IoT and knowledgeable about developing the new instrument. Thirteen experts were selected for the content validity test based on their role, skills, and experiences related to e-Government, IS, and IoT. 

This research applied a two-stage process of validation with different groups of experts, as suggested by [103,104], to exhibit high content validity of the measurement instrument. The first stage followed a qualitative assessment amongst four experts to ascertain feedback based on six questions adapted from [105]. Throughout this process, the experts provided their opinions on each construct’s measurement items and performed language editing for the instrument. This study followed an extensive and exhaustive content validity process in the first stage to clarify ambiguity and ensure that the appropriate meanings of the measurement items were conveyed. 

The second stage of this process applied a quantitative approach devised by [106], with nine experts agreeing to participate. The second stage was required when the instrument was significantly modified (i.e., several items were added to the scale in the first round) [104]. The content validity ratio (CVR) and content validity index (CVI) were used to analyse the experts’ degree of consensus to determine items’ content validity. 

The next validation stage was pre-testing. The purpose was to detect the questionnaire’s readability and any problems with structure and ambiguity issues in the survey instrument. This assessment helps the researcher avoid low data quality and item deletions during measurement model [107]. This research created the pre-test template according to the proposal by [108,109] Ten employees from LAs (with and without the knowledge of IoT) were invited to partake in a face-to-face meeting session. 

Finally, a pilot test was conducted to reduce the risk of bias and statistically determine the survey instrument’s quality before the actual data collection [100,110]. A pilot study was carried out with the respondents similar to the target population. A total of 40 bilingual questionnaires were randomly distributed through a link sent via email to the target respondents, who were Malaysian LA employees registered as IoTbs users. The list of employees was obtained from the Ministry of Urban Wellbeing and Housing. Out of 40 questionnaires, 33 were returned and used in the pilot test analysis. The results indicated that the inter-correlation among the constructs’ items was highly reliable: all constructs were higher than the threshold limit of 0.7.

### 4.3. Survey Distribution and Data Collection

The personal information available in the IoTbs database contained the registered employees’ names, departments, email addresses, and telephone numbers. Hence, an online survey appeared to be the most effective method because it could reach a large number of respondents. It was also useful in achieving high-quality data collection (i.e., the mandatory field can ensure that no missing data are received). Therefore, an online survey was the most appropriate approach for this research. The survey administration for this research commenced from 15 March 2019 to 10 May 2019. Emails were sent to the respondents with the URL link to access the survey questionnaire. 

In the first two weeks, the response rate was higher than expected (almost 60%). The surge in response might have been a side-effect of the IoTbs launching ceremony in December 2018, officiated by Malaysia’s prime minister. However, the second response rate was lower than expected. Reminders were sent via emails every two weeks following the distribution of the initial package. Specifically, the survey package was mailed on 15 March 2019. The first reminder was sent on 29 March 2019, followed by the second reminder on 12 April 2019, the third reminder on 26 April 2019, and the final reminder on 10 May 2019. To follow up on the respondents, they were contacted by phone through calls and WhatsApp messaging. The follow-up calls and messages increased the number of respondents to a total of 195 respondents. 

Initially, response rate analysis was performed to assess the research findings’ value [111]. Out of 297 survey questionnaires distributed via email to the respondents, 15 questionnaires were undelivered due to invalid email addresses and contact numbers. After 4 follow-ups, 195 online responses were received. Predictably, the response rate slightly increased after each round of follow-ups. This yielded a response rate of 69.15% (195 / (297 − 15) ∗ 100 = 69.15%). Table 2 presents a summary of the sample sizes and responses.

The demographics of both the respondents and the population were compared using chi-squared goodness-of-fit test, which had 689 employees registered as users of IoTbs in Malaysia until 2018. The list was extracted from the IoTbs database and obtained from the Ministry of Housing and Local Government. The descriptive statistics that compared the respondents to the population are displayed in Table 3. 

## 5. Results

### 5.1. Descriptive Analysis

The frequency distribution of descriptive analysis is presented in Table 4, which contains the frequencies for the number of respondents in terms of gender, age, type of service group, department and experience categories for using IoTbs. For a sample size of 187, the gender proportion of the respondents was 49.7% male and 50.3% female. The ratio between both genders was 0.6%. The majority of respondents were within the range of 31 to 40 years old (58.8%). Most of the respondents (65.8%) from the Support Group Services by the government. Furthermore, the most significant number of respondents in the sample was from the District Council (44.9%) and followed by the Municipal Council (27.8%). City Council provided the fewest respondents with a total of twenty people, which accounted for 10.7%. Meanwhile, thirty-three respondents (16.6%) were categorised as ‘Others’, whereby these respondents had not answered the question regarding work local authorities (LAs). In terms of working experience, the majority of respondents, which accounted for 62.6% of the total number of respondents, had less than five years of working experience in the government. 

### 5.2. Assessment of the Measurement Model

In measuring internal consistency, the value of true reliability of lower-order constructs (LOCs) remained between CR (representing the upper bound) and Cronbach’s alpha (representing the lower bound) [112]. However, Smart-PLS does not include measurement model statistics for HCM [113]. 

The Cronbach’s alpha and CR values for both higher-order constructs (HOCs) were calculated. Results revealed that the Cronbach’s alpha and CR for LOCs and HOCs of the model ranged between 0.787 and 0.981 and 0.731 and 0.983, respectively. These values exceeded the criterion recommended by Hair et al. [112]. Appendix A shows these results, which indicated that the measures of the construct had high internal consistency for reliability. The amount to which a measure relates to other measurements of the same phenomenon is known as convergent validity [112]. The outer loadings of indicators on the constructs for all stages of the process are presented in Appendix A, which also shows two indicators which were removed. The content of the indicator was closely examined to identify the causes for the indicator to be unreliable and to justify the consequences for content coverage. 

Appendix A shows the indicator loadings for all LOCs, and HOCs ranged between 0.747 and 0.960, which exceeded the required minimum of 0.70. High outer loadings of a construct suggested that the associated indicators had much in common, which was represented by the construct [112].Another valid option to assess convergent validity of the construct level was AVE. The AVE value for both HOCs was calculated and showed that the AVE values for ISQ in HOC and TR HOC met the threshold value (0.625 and 0.529, respectively). Appendix A displays the results that supported the convergent validity of all LOCs and HOCs.

In this study, discriminant validity was analysed through the HTMT criterion. Appendix B shows the computation that yielded values between 0.082 for HTMT (INN, DIS) and 0.970 for HTMT (USE, PU). All the LOCs had good discriminant validity because of the HTMT value significantly lower than 1. The confidence interval did not show a value of 1 on any constructs through the use of HTMT inference. In summary, all reflective measurement model evaluation criteria had been met, which supported the reliability and validity of the measures for both LOCs and HOCs (see Appendix A). 

### 5.3. Assessment of Structural Model

As described by Hair et al. [114], the results of collinearity, path coefficient, coefficient of the determinant (R^2^), effect size (f^2^), predictive relevance (Q^2^) and effect size (q^2^) were the most critical metrics for the structural model in PLS-SEM when evaluating the relationship among the constructs. Results of these analyses would be discussed below.

Collinearity can be assessed through VIF values [114]. Table 5 presents the outcome of the lateral collinearity test. All values of inner VIF for the independent variables (ISQ, PU, SN, TR, and the US) needed to be examined for lateral multicollinearity. The values obtained were less than 10, which indicated that multicollinearity was not present in this research [115].

This research developed nine direct hypotheses between the constructs and t-statistics for all paths generated using the Smart-PLS 3.0 bootstrapping function to test the significance level. The constructs of ISQ (β = 0.498, *p* < 0.01) and SN (β = 0.443, *p* < 0.01) were positively related to PU based on the path coefficient results listed in Table 6. Thus, H1 and H5 were supported. The ISQ (β = 0.490, *p* < 0.01) and PU (β = 0.454, *p* < 0.01) had a significant relationship with US, which supported H2 and H4 of this study. In terms of the determinants for IoTbs Use, PU and US showed a positive relationship, with β = 0.641, *p* < 0.01 and β = 0.333, *p* < 0.01, respectively. The results indicated that H3 and H9 were supported. Furthermore, the relationship between TR and ISQ was significant, with β = 0.746, *p* < 0.01, which supported H6. Nonetheless, TR had no significant relationship with PU (β = 0.26, *p* > 0.318) and US (β = 0.017, *p* > 0.365). Thus, H7 and H8 were rejected. To summarise, among the nine hypothesised relationships tested in this research, seven hypotheses (H1, H2, H3, H4, H5, H6 and H9) were accepted, and two hypotheses (H7 and H8) were rejected. 

**Figure 2:**
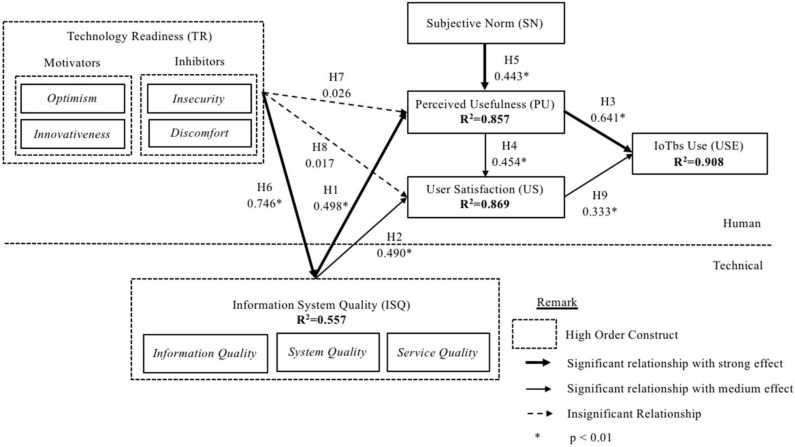
Structural model analysis results.

Results also showed that there was a significant effect on all estimated path coefficients. Figure 2 illustrates the results for the PLS-SEM structural model analysis, which indicated the path coefficient and coefficient of determination.

The amount of variance explained in IoTbs Use was 90.8% (R^2^ = 0.908). IoTbs use was directly affected by PU and US. In addition, the amount of variance explained in PU was 85.7% (R^2^ = 0.857), which was directly affected by ISQ, TR and SN. On the other hand, the US construct was directly affected by ISQ, and the amount of variance explained in US was 86.9% (R^2^ = 0.869). Similarly, IQ, SQ and SVQ directly affected ISQ, which accounted for 55.7% of the variance (R^2^ = 0.557). According to Hair et al., the amount of variance explained in all dependent variables exceeded the 75% cut-off for good explanatory power [112]. The amounts of variance explained for dependent variables in the model proved that the overall model fit was good. 

Table 7 presents the results of the effect size f^2^ for the exogenous variable based on the endogenous variable. From the table, the f^2^ of four exogenous variables had a high effect size on the respective endogenous variables (namely, ISQ on PU, PU on IoTbs Use, SN on PU and TR on ISQ). The effect size of the other three exogenous variables on the respective endogenous variable was medium (namely, ISQ on US, PU on US and US on IoTbs Use). However, there was no effect of exogenous variable TR on the endogenous variable PU and US. 

Further analysis in determining the accuracy of the model’s prediction relevance (Q^2^) was performed using the blindfolding procedure. Table 6 shows the evaluation of Q^2^ in this research. The results presented a sufficient high predictive relevance for all endogenous construct with a value between 0.375 and 0.733. Thus, this result indicated that the model had sufficient predictive relevance. 

Table 6 shows that the q^2^ effect size of SN and ISQ exogenous constructs on PU and US endogenous constructs were relatively small, except for exogenous construct ISQ on the endogenous construct PU, which reported to be medium. Other relationships such as exogenous construct TR on the endogenous construct PU, and exogenous construct TR on the endogenous construct US had no effect.

## 6. Discussion

This research developed and tested a model that explains how variables from TR, the D&M IS success model and TAM influence the use of IoTbs in FM. The results help identify the factors that affect the use of IoTbs in facilities management (FM) from employees’ perspective by focusing on the domain of Malaysian public sectors. As in quantitative research, the predicted results proves the correctness and accuracy of the proposed research model [116] A good predictive power indicates that the proposed model strikes a balance between being comprehensive and parsimonious. Detailed discussions are provided on the results obtained in this study.

### 6.1. Information System Quality 

From the technical specific aspect, the findings revealed that IS quality (ISQ) significantly affected both perceived usefulness (PU) and user satisfaction (US), which are consistent with previous studies [117,118,119]. These findings indicate that ISQ was the strongest predictor of PU and indirectly affected the use of IoTbs with a large effect size via the PU construct. To increase the level of usage of IoTbs in FM by employees, the overall quality of the IoTbs is critical to gain employees’ satisfaction and to demonstrate the usefulness of the service. The overall quality of IoTbs in terms of ease, timeliness, accuracy, and responsiveness when making a proper predictive maintenance analysis based on facilities conditions may make employees feel that using IoTbs is useful, which, in turn, make them feel satisfied; thus, they are willing to use IoTbs. 

### 6.2. Technology Readiness 

This research confirmed the impact of TR on the success of IoTbs. The present research found the significant effects of TR on overall ISQ in the technical aspect with substantial effect size. The result indicates that employees with higher TR generally evaluate a technology more highly regarding the overall quality of IoTbs, which then increases their usage. This research clarified that employees with a positive view of technology generally did not focus on negative aspects of the quality of IoTbs (such as inconsistency and incompleteness of the information). They are more likely to confront the overall quality of IoTbs more openly, and positively, hence increasing the usage.

The current results further show that TR was an insignificant factor because it neither influenced the employees’ perception of usefulness nor the employees’ satisfaction. Apparently, individual’s personality differences potentially influence how employees form their perception towards use behaviour. This means that IoTbs users have different personalities from users of other technologies, such as self-service technologies (SSTs) or online services. IoTbs users have more negative feelings in using IoTbs.

These insignificant results may be due to the relationship between TR and PU, and 80% of the respondents in this research being Generation Y, i.e., born in the 1980s and 1990s. Previous studies have claimed that Generation Y employees are typically familiar with electronic and digital technology [120] and tend to have strong positive work attitudes [121] Employees with positive work attitudes have greater involvement and engagement in work [122], as they will always perceive the new initiative proposed by the government is useful. 

### 6.3. Subjective Norm

A large effect size has been obtained on the association between SN and PU. The findings of this research also show that employees are affected by evaluations and recommendations from their colleagues and supervisors to perceive IoTbs as useful. Regarding IoTbs, these findings aligned with prior findings in the case of IoTbs in smart cities in China [123] and smart devices in India [84]. The present study also confirmed the findings by Venkatesh and Davis [73], who firstly proposed the effects of SN on PU. In line with their views, the present research provides support to the belief that if a supervisor or co-worker recommends that a certain system would be useful, the person may start to accept that it is useful, and develop a habit of using it. When it comes to using IoTbs in FM, employees influence each other’s beliefs about the usefulness of IoTbs in FM, which increases the likelihood of using IoTbs in FM. 

### 6.4. Perceived Usefulness

As a human factor, the perception of the usefulness of IoTbs affects both the IoTbs Use and US. The strength of the relationship between PU and IoTbs Use was much greater than the relationship between PU and US. It is evident from the analysis of this research that employees would feel satisfied if they felt that the services enhanced their work performance and helped them accomplish their work tasks efficiently and smoothly. Simultaneously, the results suggested that employees were likely to use IoTbs when they perceived IoTbs to be useful. IoTbs can add benefit to employees and greatly enhance their perceived value. These findings confirmed that PU should be considered as a key to IoTbs success measure, specifically in increasing the use of IoTbs in FM. Therefore, the government should consider these factors to increase the utilisation of IoTbs among employees. Meanwhile, application developers and service providers should also consider these factors to increase users’ desire to use IoTbs.

### 6.5. User Satisfaction

US was found to affect IoTbs Use with moderate effect size. It can be construed that the high utilisation of IoTbs Use is due to employees’ high satisfaction levels. These empirical results also show that the ISQ and PU had a statistically significant influence on US. It can be interpreted as a response to the two types of employees’ expectations about IoTbs: they want their IoTbs to be of high quality and provide a high perception of the usefulness. Overall, this result suggests that although the degree of employees’ satisfaction significantly impacts their utilisation of IoTbs, their perception of usefulness is more critical for IoT success than their satisfaction. Employees who find using IoTbs useful may lead to their decisions to use the system although they might discover particular issues that might affect their satisfaction. 

### 6.6. IoTbs Use

In this research, technical and human factors have been introduced into the nomological network related to IoTbs Use, thus expanding the understanding of the phenomenon of IoT success. This research theorized and found that technical and human factors are strongly related to the use of IoTbs in FM. Moreover, this research is one of the first studies in the IS success literature that integrates and tests empirically TR and SN as an additional predictor of IoT success. The findings of the present research provide strong predictive power of the success model for IoTbs in FM, explaining 90% of the variance in IoTbs Use compared with previous research, which was only 53% according to [21]. This research confirms the assertion of previous researchers that the system use is appropriate to be an indicator of success [66,124,125,126,127].

## 7. Research Contributions

### 7.1. The IoT-Based Services Success Model 

To determine the contributions of this research to theory, the IoTbs success model established in this research was compared with a previous study on IoT success that mainly relied on the extended version of D&M IS success model. Table 8 compares the contexts and setting of the studies. To the best of our knowledge, this research and the study by [21] are the only studies which have been conducted in the IoT context. However, Chatterjee et al. (2018) performed their study with a different perspective of the IoT. Their research aimed to develop an IoT success model in India’s smart cities, whereas this research focused on the development of the IoT success model in FM. 

In contrast with other success models, this is the first successful model that considers the human factors in addition to technical factors from perspectives of personality traits of individuals and social influences, known as TR and SN. TR and SN, which have recently gained considerable attention among researchers in evaluating new emerging technologies, were previously used in acceptance models. However, the roles of TR and SN showed little evidence in previous studies of success of IoTbs in the context of the public sector, particularly from employees’ perspectives. Therefore, this study provides more evidence on the needs of having TR and SN in measuring the success of IoTbs. This study was also the first to develop and examine an IoTbs success model that included IoTbs Use as a nature of the use of the dependent variable. Finally, an integrated IoTbs success model in FM was developed to address the human and technical factors, which were the limitations of the existing studies. 

The present research enhances the D&M IS success model through the combination of TRI and other variables from other theories; hence responding to a call by Delone and Mclean [125] to extend the D&M IS success model to include other success measures when evaluating the success of emerging technologies. This study demonstrates the applicability of the D&M IS success model not only in traditional ISs, but also in the latest trends of technology, hence justifying the D&M IS success model as a parsimonious framework for predicting and explaining individuals’ use of various technologies.

### 7.2. The Technology Readiness and Subjective Norm in IoTbs Success Model

This research aimed to understand TR’s construct by investigating its dimensionality and assessing TR’s effect on IoT usage. Theoretically, this research integrated TR into the D&M IS success model to enhance understandings of TR, investigate its impacts on IoT usage, and provide a holistic view of IoTbs success. Although previous studies have examined the impacts of TR by extending the technology acceptance model (TAM) [89,128,129], the impacts of TR from employees’ perspectives have largely been ignored in explaining use behaviour towards IoTbs success. Thus, the present research extends the D&M IS success model by adding TR and has tested its applicability within the context of IoTbs in FM. 

In addition, this research contributes to the conceptualisation of the constructs of TR. The analysis of present research clarified that the construct is best conceptualised as a one-dimensional higher-order construct (HOC). This conceptualisation had the best model fit and should be used in IoT research because it outperforms alternative conceptualisations. Finally, the inclusion of TR in the present model further extends the applicability of the D&M IS success model, hence responding to a call for additional theory-testing efforts to validate research results accumulated from prior studies on D&M IS success model. 

From a theoretical perspective, the inclusion of subjective norms in the present model further enhances the validity of the findings of existing studies that support the effect of SN on PU in determining the use of technologies. As evident from the literature, the role of social influence or SN constructs has been controversial. Some have argued for their inclusion in models of adoption and use (e.g., whereas others have not included them (e.g., Davis, 1989a; Chatterjee et al. (2018). The present research suggests that subjective norms do matter, and shed light on the importance of SN in driving behaviour of employees, particularly on the success of IoTbs.

### 7.3. Methodological Contributions

The methodological contribution of the current study can be seen as threefold. Currently, there are limited studies applying a PLS marker variable as an alternative approach to diagnose and control the problems associated with common method variance (CMV). Therefore, this research contributes to the methodological approach of PLS marker variable. The results indicate that the PLS marker variable approach is a practical and useful alternative for controlling a CMV. 

The second contribution to consider is the implementation of the two-stage approach during content validation. The initial stage involved a qualitative assessment among subject matter experts to seek their feedback on the instruments. Based on the feedback, the instrument items were revised and modified accordingly. The next stage involved a quantitative assessment, where the SMEs were invited to choose the most accurate and critical content of the instruments through the measurements of the content validity ratio, content validity index, and mean score. The two-stage approach was applied to propose a reliable and valid instrument of the IoT success model in FM. The assessment method of content validation in this study further enhances the maturation of the validation guidelines for positivist IS studies.

## 8. Implications 

### 8.1. Governments

The involvement of employees in daily improvements is critical for successful IoT implementation and IoT talent development. To ensure that a country has a ready pool of competent IoT talent in all domains, there is a need for governments to improve their strategic plans and guidelines concerning individual’s perceptions of technology. For local agencies, they may promote the increasing use of IoTbs in FM by taking into account employees’ personality traits in encouraging them to use IoTbs. Technology readiness has attributes of drivers (optimism and innovativeness) and inhibitors (discomfort and insecurity); therefore, local agencies that plan to use IoTbs in FM may have the strategies for drivers and inhibitors. Although IoTbs may attract people who are optimistic and innovative, IoTbs may hinder those who are not comfortable and insecure in using them. TR had a significant effect on the overall quality of the IoTbs; therefore, local agencies may need to ensure that the system quality, information quality and service quality of IoTbs in FM are excellent to attract those who are optimistic and innovative and to encourage people who are discomfort and insecure. When the overall quality of the IoTbs in FM is excellent, the employees will feel more comfortable and secure in using the IoTbs in FM. 

There was a significant effect of SN to PU; therefore, local agencies may strategize ways to improve the peer relationships. Among the recommendations are to organise social networking among communities of local agencies so that the usefulness of the IoTbs can attract more employees to use IoTbs by word of mouth. Additionally, local agency managers should focus on encouraging personnel with high position or influence to adopt IoTbs early in the organization’s implementation.

### 8.2. IoT Developers

The results of this research indicate the importance of the overall quality of IoTbs in terms of system quality, information quality and service quality. IoT developers have to ensure that the overall quality of IoTbs considers the diversity of employees, who could be optimistic and innovative and who not comfortable and insecure. Therefore, understanding the personality traits of employees may guide the developers in improving the development of IoTbs. These factors may inspire the developers to design interfaces and functions that offer better characteristics of the services’ output and performance, which may increase employees’ satisfaction and their perceptions of the IoTbs usefulness.

## 9. Limitations and Future Work 

When generalising the results obtained from this research, several limitations that point to future research are notable due to the research participants’ options, model, and approach. One of the limitations is that the measurement of the constructs typically involved in the self-reported answer. Hence, response biases could occur because the accuracy of answers given was obtained by depending on the respondents’ readiness and ability to present the right responses. Another limitation of this research is that the study only relied on cross-sectional data and cannot directly evaluate effects over time. 

Based on the contributions and limitations, this research provides some recommendations for future research. A significant limitation of this research is its reliance on quantitative survey research. Hence, it did not interpret and capture rich data. However, conducting controlled experiments at every local authority (particularly in FM departments) around Malaysia could be difficult because of the ethical and cost considerations. Thus, future research could further explore this research’s effects in three ways: (1) using a qualitative approach; (2) using a mixed-method approach; or (3) using expert review. Particularly for deep insights, all approaches are best when a phenomenon is vague. There is a need to be understood because it is a new topic, or only little research has investigated it. Future research could more specifically address the research question of how local authorities’ employees deal with the implementation of IoTbs. 

After the COVID-19 pandemic, things have changed, especially the engagement and behaviours with online systems. Hence, there is a need to re-examine the proposed model after the pandemic to compare the findings and determine how the pandemic has impacted e-services.

## 10. Conclusions

This research aimed to investigate the factors that influence individuals to use IoTbs in FM, with success is interpreted as IoTbs use. The research model was developed based on the D&M IS success model. A cross-sectional survey design was performed to test the proposed model. The research respondents were public employees who were using IoTbs. A set of research hypotheses was then empirically tested using PLS-SEM.

This research established the validity of the D&M IS success model [130] in the context of IoT use in FM. This research found the importance of inserting technical and human factors in models of IoT success. TR as a human factor via ISQ and SN was found to have strong significant effects on PU, which is consistent with prior studies. TR via ISQ was also found to have strong significant effects on US. On the other hand, ISQ as the technical factor as a strong total effect on IoTbs Use. This research confirmed the validity of the D&M IS success model in the context of IoTbs utilisation and the importance of including other related factors. The important implications for practice were discussed, and several future research avenues were recommended based on the findings of this research. 

## Figures and Tables

**Table 1:** Scope of IoTbs for FM.

Sources	Country	Scope of FM	IoT Devices/Tools
[3]	Malaysia	The scope is complete, including landscaping services, cleaning services, mechanical and electrical services, civil services and many others.	GPS, sensors, smartphone, Global Navigation Satellite System (GNSS), cloud computing.
[36]	Finland	Building information modelling	Sensor readings, Big Data analysis, connected sensor networks, ubiquitous computing
[37]	Sweden	Inclusive of invisible services, business infrastructure management and asset management for buildings.	Artificial intelligence (AI), smartphone, sensors.
[38]	Australia	It is related to buildings and infrastructure that depend on a slew of incompatible technologies to keep track of asset value management and building maintenance.	RFID, sensors, laser scanning, wireless sensor networks, smartphone Bluetooth, webcam-enabled handheld devices.
[39]	China	Facilities’ energy consumption, the security of facilities, the quality and ambient comfort of the indoor environment, the assessment of infrastructures, visualisation of facilities’ information, the use of interior space, and structural health.	Auto-ID, sensor, photogrammetry/videogrammetry and laser scanning, wireless sensor networks (WSNs).

**Table 2:** Response rate.

Results	N *
Total sent	297
Undeliverable because of a non-valid email address and contact number	−15
Responses after the first email	117
Responses after the second reminder	28
Responses after the third reminder	23
Responses after the fourth reminder	27
Total usable returned questionnaires	195

* N, number of questionnaires.

**Table 3:** Comparing respondents with the population.

	Respondents		Population	
Female	98	50.3%	373	54.1%
Male	97	49.7%	316	45.9%

**Table 4:** Frequency distribution of descriptive analysis.

Demographic Attribute	Category	Frequency	Percentage (%)	Valid Percentage (%)	Cumulative Percentage (%)
Gender	Male	93	49.7	49.7	49.7
	Female	94	50.3	50.3	100.0
Age (years)	18–30	40	21.4	21.4	21.4
	31–40	110	58.8	58.8	80.2
	41–50	27	14.4	14.4	94.7
	>51	10	5.3	5.3	100.0
Education Level	SPM or SPTM or SPMV	27	14.4	14.4	14.4
	Diploma	60	32.1	32.1	46.5
	Bachelor’s degree	81	43.3	43.3	89.8
	Master’s degree	14	7.5	7.5	97.3
	Doctorate	1	.05	0.5	97.9
	Others	4	2.1	2.1	100.0
Service Group	Support Group	123	65.8	65.8	65.8
	Management and Professional	59	31.6	31.6	97.3
	Top-Level Management	5	2.7	2.7	100.0
Department	District Council	84	44.9	44.9	44.9
	Municipal Council	52	27.8	27.8	72.7
	City Council	20	10.7	10.7	83.4
	Others	31	16.6	16.6	100.0
Working Experience (years)	<5	117	62.6	62.6	62.6
	>5	70	37.4	37.4	100.0

**Table 5:** Collinearity analysis result.

Construct	PU	IoTbs Use	US
ISQ	4.028		5.544
PU		5.537	5.215
SN	3.991		
TR	2.504		2.357
US		5.537	

**Table 6:** Path coefficient result.

Hypothesis	Relationship	Std. Beta	Std. Error	T-Value	*p*-Value	Decision
H1	ISQ→PU	0.498	0.079	6.311	0.000	Supported
H2	ISQ→US	0.490	0.077	6.347	0.000	Supported
H3	PU→IoTbs Use	0.641	0.081	7.910	0.000	Supported
H4	PU→US	0.454	0.077	5.861	0.000	Supported
H5	SN→PU	0.443	0.091	4.877	0.000	Supported
H6	TR→ISQ	0.746	0.035	21.154	0.000	Supported
H7	TR→PU	0.026	0.056	0.472	0.318	Not Supported
H8	TR→US	0.017	0.050	0.344	0.365	Not Supported
H9	US→IoTbs Use	0.333	0.085	3.916	0.000	Supported

**Table 7:** Summary of R^2^, f^2^ Q^2^, and q^2^ results.

Hypothesis	Relationship	R^2^	f^2^	Q^2^	q^2^
H1	IS Quality→Perceived Usefulness	0.857	0.434	0.714	0.182
H2	IS Quality→User Satisfaction		0.331		0.135
H3	Perceived Usefulness→Use	0.908	0.808	0.716	
H4	Perceived Usefulness→User Satisfaction	0.869	0.303	0.733	
H5	Subjective Norm→Perceived Usefulness		0.342		0.143
H6	TR→IS Quality	0.558	1.261	0.375	
H7	TR→Perceived Usefulness		0.002		0.000
H8	TR→User Satisfaction		0.001		−0.004
H9	User Satisfaction→Use		0.217		

**Table 8:** A comparison between the present research and prior research of IoT success—study settings.

Study	Industry and Country: Unit of Analysis (Respondent)	Types of IoT/IS (Dependent Variable)
The present research	Facilities Management in Malaysia: Individual (Public Employees’)	IoT-based Facilities Management (IoTbs use)
[21]	Smart Cities in India: Individual (Citizens)	General IoT devices used for Smart Cities (Actual usage of IoT)

## Data Availability

Data are available from the corresponding author for researchers who meet the criteria for access the data.

## Appendix A. Summary of Measurement Model Analysis

**Construct**		**Indicators**	**Convergent Validity**		**Internal Consistency**		**Discriminant Validity**
**Name**	**Type**		**Outer Loadings**	**AVE**	**Cronbach’s Alpha**	**Composite Reliability**	**HTMT ***
			**>0.70**	**>0.50**	**>0.70**	**>0.70**	
TRI	HOC	DIS	-	0.529	0.953	0.869	Yes
		INN	-				
		INS	-				
		OPT	-				
Discomfort	LOC	DIS2	0.788	0.701	0.787	0.875	Yes
		DIS3	0.879				
		DIS4	0.843				
Innovativeness	LOC	INN1	0.802	0.652	0.822	0.882	Yes
		INN2	0.747				
		INN3	0.852				
		INN4	0.824				
Insecurity	LOC	INS2	0.861	0.700	0.786	0.875	Yes
		INS3	0.849				
		INS4	0.798				
Optimism	LOC	OPT1	0.921	0.843	0.938	0.955	Yes
		OPT2	0.919				
		OPT3	0.907				
		OPT4	0.925				
IS Quality	HOC	IQ	-	0.625	0.984	0.974	Yes
		SQ	-				
		SVQ	-				
Information Quality	LOC	IQ1	0.871	0.768	0.962	0.968	Yes
		IQ2	0.889				
		IQ3	0.896				
		IQ4	0.911				
		IQ5	0.870				
		IQ6	0.893				
		IQ7	0.797				
		IQ8	0.856				
		IQ9	0.901				
		IQ10	0.877				
System Quality	LOC	SQ1	0.896	0.795	0.957	0.964	Yes
		SQ2	0.889				
		SQ3	0.927				
		SQ5	0.883				
		SQ6	0.869				
		SQ7	0.868				
		SQ8	0.907				
Service Quality	LOC	SVQ1	0.830	0.808	0.940	0.954	Yes
		SVQ2	0.938				
		SVQ3	0.928				
		SVQ4	0.908				
		SVQ5	0.885				
		SVQ6	0.839				
Perceived Usefulness	LOC	PU1	0.936	0.896	0.971	0.977	Yes
		PU2	0.955				
		PU3	0.959				
		PU4	0.941				
		PU5	0.941				
Subjective Norm	LOC	SN1	0.957	0.919	0.911	0.958	Yes
		SN2	0.960				
User Satisfaction	LOC	US1	0.945	0.902	0.964	0.974	Yes
		US2	0.957				
		US3	0.949				
		US4	0.948				
Use	LOC	USE1	0.938	0.853	0.971	0.976	Yes
		USE2	0.939				
		USE3	0.941				
		USE4	0.920				
		USE5	0.937				
		USE7	0.912				
Note: * HTMT confidence interval does not include 1.

## Appendix B. Validity Result using HTMT Criterion

**Construct**	**DIS**	**IQ**	**INN**	**INS**	**OPT**	**PU**	**SVQ**	**SN**	**SQ**	**USE**	**US**
Discomfort (DIS)											
Information Quality (IQ)	0.223 CI.90 (0.097, 0.369)										
Innovativeness (INN)	0.082 CI.90 (0.041, 0.066)	0.625 CI.90 (0.525, 0.720)									
Insecurity (INS)	0.637 CI.90 (0.502, 0.769)	0.212 CI.90 (0.093, 0.330)	0.134 CI.90 (0.068, 0.170)								
Optimism (OPT)	0.146 CI.90 (0.049, 0.279)	0.743 CI.90 (0.667, 0.805)	0.757 CI.90 (0.664, 0.819)	0.123 CI.90 (0.045, 0.214)							
Perceived Usefulness (PU)	0.202 CI.90 (0.096, 0.338)	0.863 CI.90 (0.783, 0.916)	0.619 CI.90 (0.525, 0.712)	0.179 CI.90 (0.066, 0.311)	0.757 CI.90 (0.676, 0.812)						
Service Quality (SVQ)	0.246 CI.90 (0.111, 0.399)	0.898 CI.90 (0.840, 0.942)	0.603 CI.90 (0.504, 0.707)	0.200 CI.90 (0.086, 0.326)	0.730 CI.90 (0.663, 0.783)	0.903 CI.90 (0.863, 0.936)					
Subjective Norm (SN)	0.258 CI.90 (0.121, 0.409)	0.853 CI.90 (0.790, 0.917)	0.635 CI.90 (0.543, 0.727)	0.182 CI.90 (0.071, 0.322)	0.805 CI.90 (0.750, 0.859)	0.941 CI.90 (0.899, 0.983)	0.896 CI.90 (0.849, 0.943)				
System Quality (SQ)	0.197 CI.90 (0.097, 0.338)	0.944 CI.90 (0.902, 0.970)	0.63 CI.90 (0.536, 0.714)	0.201 CI.90 (0.095, 0.321)	0.767 CI.90 (0.703, 0.821)	0.928 CI.90 (0.897, 0.949)	0.958 CI.90 (0.921, 0.976)	0.896 CI.90 (0.849, 0.939)			
IoTbs Use (USE)	0.192 CI.90 (0.094, 0.327)	0.868 CI.90 (0.817, 0.904)	0.652 CI.90 (0.567, 0.731)	0.151 CI.90 (0.055, 0.277)	0.760 CI.90 (0.699, 0.811)	0.970 CI.90 (0.953, 0.991)	0.909 CI.90 (0.873, 0.939)	0.948 CI.90 (0.915, 0.990)	0.914 CI.90 (0.872, 0.941)		
User Satisfaction (US)	0.175 CI.90 (0.070, 0.324)	0.889 CI.90 (0.837, 0.930)	0.610 CI.90 (0.525, 0.708)	0.174 CI.90 (0.064, 0.307)	0.749 CI.90 (0.686, 0.807)	0.935 CI.90 (0.894, 0.963)	0.926 CI.90 (0.885, 0.952)	0.899 CI.90 (0.855, 0.951)	0.933 CI.90 (0.901, 0.957)	0.943 CI.90 (0.911, 0.967)	
Note: The value in brackets represent the 95% bias-corrected and accelerated confidence interval of the HTMT values obtained by running the bootstrapping routine with 5000 samples in Smart-PLS.

## Appendix C. Questionnaire

QUESTIONNAIRE/*SOAL SELIDIK*

Successful Internet of Things-based Services (IoTbs) in Facilities Management Among Local Authorities’ Employees.

Kejayaan Perkhidmatan Pintar berasaskan Internet of Things (IoTbs) bagi Pengurusan Fasiliti di Kalangan Pekerja Pihak Berkuasa Tempatan.

Internet of Things based Services (IoTbs)—information technology and information systems you use in your current job for work integration to plan and coordinate work activities, monitor work performance, and communicate with superiors and subordinates. Some technologies relevant to IoTbs are real-time location tracking and navigation using satellite technology. IoTbs may also provide real-time push notification using Firebase Cloud Messaging (FCM), key performance indicator (KPI) alerts, and smartphone application controls using mobile cloud computing. The IoTbs example being implemented by the Government is iTegur, KPKT.

Perkhidmatan Pintar berasas Internet of Things (IoTbs)—teknologi maklumat dan sistem maklumat yang anda guna dalam tugas semasa anda untuk integrasi kerja bagi merancang dan menyelaras aktiviti kerja, memantau prestasi kerja, dan berkomunikasi dengan pegawai atasan dan bawahan. Antara teknologi yang berkaitan dengan IoTbs adalah pengesanan dan navigasi lokasi masa nyata menggunakan teknologi satelit, notifikasi pemberitahuan masa nyata menggunakan Firebase Cloud Messaging (FCM), notifikasi petunjuk prestasi utama (PPU), dan kawalan aplikasi telefon pintar mengguna pengkomputeran awan mudah alih. Contoh IoTbs yang sedang dilaksana Kerajaan adalah iTegur, KPKT.

Please evaluate the importance of each item by ticking as appropriate.

Sila menilai perkara penting bagi setiap item dengan menanda pada pilihan yang bersesuaian.

### Appendix C.1. Technology Readiness/Seksyen 1: Kesediaan Teknologi

This section asks your perception about employees’ mental readiness to accept new technology that utilise IoTbs.

Seksyen ini menanya pandangan anda mengenai kesediaan mental pekerja untuk menerima teknologi baru yang mempengaruhi penggunaan IoTbs.

**No.**	**Item**	**1** **Strongly Disagree** ***Sangat Tidak Bersetuju***	**2** **Disagree** ***Tidak Bersetuju***	**3** **Slightly** **Disagree** ***Kurang Bersetuju***	**4** **Neutral** ***Neutral***	**5** **Slightly** **Agree** ***Sedikit* *Bersetuju***	**6** **Agree** **Bersetuju**	**7** **Strongly Agree** ***Sangat Bersetuju***
**OPT1**	**IoTbs contribute to a better quality of work.** *IoTbs menyumbang kepada kualiti kerja yang lebih baik.*	□	□	□	□	□	□	□
	**IoTbs gives or makes me…** *IoTbs memberi atau membuat saya…*							
**OPT2**	**more freedom of mobility.** *lebih kebebasan untuk bergerak.*	□	□	□	□	□	□	□
**OPT3**	**more control over my daily work.** *lebih kawalan terhadap kerja harian saya.*	□	□	□	□	□	□	□
**OPT4**	**more productive in my work.** *lebih produktif dalam kerja saya.*	□	□	□	□	□	□	□
**INN1**	**Other officers come to me for advice on new technologies.** *Pegawai lain datang kepada saya untuk mendapat nasihat berkenaan teknologi baru.*	□	□	□	□	□	□	□
**INN2**	**I can usually figure out new high-tech products and services without help from others.** *Saya selalunya boleh mencari produk dan perkhidmatan berteknologi tinggi tanpa bantuan orang lain.*	□	□	□	□	□	□	□
**INN3**	**I keep up with the latest technological developments in my areas of interest.** *Saya sentiasa mengambil tahu pembangunan teknologi terkini dalam bidang yang saya minati.*	□	□	□	□	□	□	□
**INN4**	**In general, I am among the first in my circle of friends to acquire new technology when it appears.** *Secara umumnya, saya adalah antara orang yang pertama dikalangan rakan saya yang memerlukan teknologi baru apabila ia muncul.*	□	□	□	□	□	□	□
**DIS1**	**When I get technical support from a provider of IoTbs, I sometimes feel as if I am being taken advantage of by someone who knows more than I do.** *Semasa saya menerima khidmat sokongan teknikal daripada pembekal IoTbs, saya kadang-kala berasa seperti saya sedang diambil kesempatan oleh seseorang yang lebih berpengetahuan daripada saya.*	□	□	□	□	□	□	□
**DIS2**	**The technical support team are not helpful because they do not explain things in terms I understand.** *Pasukan sokongan teknikal tidak membantu kerana mereka tidak menjelas sesuatu dalam perkataan yang saya faham.*	□	□	□	□	□	□	□
**DIS3**	**Sometimes, I think that IoTbs are not designed for use by ordinary people.** *Kadang kala, saya berasa bahawa IoTbs tidak direka bentuk untuk kegunaan orang biasa.*	□	□	□	□	□	□	□
**DIS4**	**There is no such thing as a manual for IoTbs are written in plain language.** *Tidak wujud sesuatu sebagai manual bagi IoTbs yang ditulis dalam bahasa yang mudah difahami.*	□	□	□	□	□	□	□
**INS1**	**Officers are too dependent on IoTbs to do things for them.** *Pegawai terlalu bergantung pada IoTbs bagi melakukan sesuatu untuk mereka.*	□	□	□	□	□	□	□
**INS2**	**Too much technology distracts officer to the point that is harmful.** *Teknologi yang terlalu banyak mengganggu pegawai sehingga ke tahap bahaya.*	□	□	□	□	□	□	□
**INS3**	**IoTbs decrease the quality of relationships by reducing personal interaction.** *IoTbs merendah kualiti hubungan dengan mengurang interaksi personal.*	□	□	□	□	□	□	□
**INS4**	**I do not feel confident doing work at a place that can only be reached online.** *Saya tidak berasa yakin melakukan kerja di tempat yang hanya boleh dicapai secara dalam talian.*	□	□	□	□	□	□	□

### Appendix C.2. Overall Information System Quality/Seksyen 2: Kualiti Keseluruhan Sistem Maklumat

This section asks your perception about the overall quality of IoTbs, including data quality, system quality and service quality.

Seksyen ini menanya pandangan anda mengenai keseluruhan kualiti IoTbs yang merangkumi kualiti data, kualiti sistem dan kualiti perkhidmatan.

**No.**	**Item**	**1** **Strongly Disagree** ***Sangat Tidak Bersetuju***	**2** **Disagree** ***Tidak Bersetuju***	**3** **Slightly** **Disagree** ***Kurang Bersetuju***	**4** **Neutral** ***Neutral***	**5** **Slightly** **Agree** ***Sedikit* *Bersetuju***	**6** **Agree** **Bersetuju**	**7** **Strongly Agree** ***Sangat Bersetuju***
	**Information provided by IoTbs …** *Maklumat yang disedia oleh IoTbs…*							
**IQ1**	**is accurate.** *adalah tepat.*	□	□	□	□	□	□	□
**IQ2**	**is up to date.** *adalah terkini.*	□	□	□	□	□	□	□
**IQ3**	**is in a useful format.** *adalah dalam format yang berguna.*	□	□	□	□	□	□	□
**IQ4**	**is clear.** *adalah jelas.*	□	□	□	□	□	□	□
**IQ5**	**meets my needs.** *memenuhi keperluan saya.*	□	□	□	□	□	□	□
**IQ6**	**is reliable.** *adalah boleh dipercayai.*	□	□	□	□	□	□	□
**IQ8**	**returns answer to my request quickly.**	□	□	□	□	□	□	□
**IQ9**	**produces comprehensive information.** *memberi maklumat yang komprehensif.*	□	□	□	□	□	□	□
**IQ10**	**In general, IoTbs provide me with high-quality information.** *Secara keseluruhan, IoTbs memberi saya maklumat yang berkualiti tinggi.*	□	□	□	□	□	□	□
	**IoTbs …** *IoTbs …*	□	□	□	□	□	□	□
**SQ1**	**is easy to use.** *adalah senang diguna.*	□	□	□	□	□	□	□
**SQ2**	**performs reliably.** *beroperasi dengan lancar.*	□	□	□	□	□	□	□
**SQ3**	**makes information easy to access.** *boleh mencapai maklumat dengan mudah*	□	□	□	□	□	□	□
**SQ5**	**can be adapted to meet a variety of needs.** *boleh diadaptasi bagi memenuhi pelbagai keperluan.*	□	□	□	□	□	□	□
**SQ6**	**offers appropriate functionality.** *menawar fungsi yang bersesuaian.*	□	□	□	□	□	□	□
**SQ7**	**The response time (refers to the elapsed time for terminal type request or entry) of IoTbs is acceptable.** *Masa maklum balas (merujuk kepada masa berlalu bagi permintaan atau kemasukan jenis terminal) IoTbs adalah boleh diterima.*	□	□	□	□	□	□	□
**SQ8**	**Overall, IoTbs is of a high-quality system** *Keseluruhannya, IoTbs adalah sistem berkualiti tinggi.*	□	□	□	□	□	□	□
	**IoTbs provider…** *Penyedia perkhidmatan IoTbs…*	□	□	□	□	□	□	□
**SVQ1**	**has up-to-date hardware and software.** *mempunyai perkakasan dan perisian yang terkini.*	□	□	□	□	□	□	□
**SVQ2**	**is dependable.** *adalah boleh diharap.*	□	□	□	□	□	□	□
**SVQ3**	**give prompt service to users.** *memberi perkhidmatan segera kepada pengguna.*	□	□	□	□	□	□	□
**SVQ4**	**has the knowledge to do their job well.** *mempunyai pengetahuan bagi melaksana kerja mereka dengan baik.*	□	□	□	□	□	□	□
**SVQ5**	**has users’ best interests at heart.** *mengambil berat tentang kepentingan pengguna.*	□	□	□	□	□	□	□
**SVQ6**	**In general, IoTbs provide me with high-quality technical support services.** *Secara keseluruhan, IoTbs memberi saya khidmat sokongan teknikal yang berkualiti tinggi.*	□	□	□	□	□	□	□

### Appendix C.3. Success Factor/Seksyen 3: Faktor Kejayaan

This section asks your perception about the level of satisfaction towards user’s experience of the IoTbs, the degree to which a person believes that using an IoTbs would enhance their job performance and the important people to you influence use behaviour towards IoTbs.

Seksyen ini menanya pandangan anda mengenai tahap kepuasan terhadap pengalaman mengguna IoTbs, sejauh mana seseorang percaya bahawa penggunaan IoTbs akan meningkat prestasi kerja mereka dan individu yang penting kepada anda mempengaruhi tingkah laku penggunaan terhadap IoTbs.

**No.**	**Item**	**1** **Strongly Disagree** ***Sangat Tidak Bersetuju***	**2** **Disagree** ***Tidak Bersetuju***	**3** **Slightly** **Disagree** ***Kurang Bersetuju***	**4** **Neutral** ***Neutral***	**5** **Slightly** **Agree** ***Sedikit* *Bersetuju***	**6** **Agree** **Bersetuju**	**7** **Strongly Agree** ***Sangat Bersetuju***
	**I am satisfied with IoTbs …** *Saya puashati dengan ____ IoTbs*							
**US1**	**efficiency.** *kecekapan.*	□	□	□	□	□	□	□
**US2**	**effectiveness.** *keberkesanan.*	□	□	□	□	□	□	□
**US3**	**I am satisfied that IoTbs meet my knowledge or information processing needs.** *Saya puashati bahawa IoTbs memenuhi pengetahuan saya atau keperluan pemprosesan maklumat.*	□	□	□	□	□	□	□
**US4**	**Overall, I am satisfied with IoTbs.** *Secara keseluruhan, saya puashati dengan IoTbs.*	□	□	□	□	□	□	□
		□	□	□	□	□	□	□
	**I use this IoTbs to…** *Saya guna IoTbs ini untuk...*	□	□	□	□	□	□	□
**USE1**	**help me manage my work.** *membantu saya mengurus kerja.*	□	□	□	□	□	□	□
**USE2**	**monitor my own performance.** *memantau prestasi kerja saya.*	□	□	□	□	□	□	□
**USE3**	**plan my work.** *merancang kerja saya.*	□	□	□	□	□	□	□
**USE4**	**get feedback on job performance.** *mendapat maklum balas mengenai prestasi kerja.*	□	□	□	□	□	□	□
**USE5**	**keep my supervisor informed.** *memasti penyelia saya maklum.*	□	□	□	□	□	□	□
**USE7**	**communicate with people who report to me.** *berkomunikasi dengan pegawai bawahan saya.*	□	□	□	□	□	□	□
**USE8**	**communicate with who I report to.** *berkomunikasi dengan pegawai atasan saya.*	□	□	□	□	□	□	□
	**Using IoTbs…** *Mengguna IoTbs akan…*	□	□	□	□	□	□	□
**PU1**	**improves my job performance.** *meningkat prestasi kerja saya.*	□	□	□	□	□	□	□
**PU2**	**makes it easier to do my job.** *memudah melaksana kerja.*	□	□	□	□	□	□	□
**PU3**	**enhances my effectiveness on the job.** *meningkat tahap keberkesanan kerja saya.*	□	□	□	□	□	□	□
	**Using IoTbs in my job…** *Mengguna IoTbs dalam kerja saya akan…*	□	□	□	□	□	□	□
**PU4**	**enables me to accomplish tasks more quickly.** *memboleh saya menyiap tugas dengan cepat.*	□	□	□	□	□	□	□
**PU6**	**I find IoTbs useful in my job.** *Saya merasa IoTbs berguna dalam kerja saya.*	□	□	□	□	□	□	□
	**People who…** *Orang yang …*	□	□	□	□	□	□	□
**SN1**	**influence me think I should use the IoTbs.** *mempengaruhi saya berpendapat bahawa saya harus mengguna IoTbs.*	□	□	□	□	□	□	□
**SN2**	**are important to me think I should use the IoTbs.** *penting bagi saya berpendapat bahawa saya harus mengguna IoTbs.*	□	□	□	□	□	□	□

### Appendix C.4. Profile of The Participant/Seksyen4: Profil Peserta

This section asks about your demographic information.

Seksyen ini adalah berkaitan maklumat demografik anda.

**L1**	**Gender:** *Jantina:* □ **Male**/*Lelaki* □ **Female**/*Perempuan*	**L2**	**Age:** *Umur:* □ **18–30 years**/*18-30 tahun* □ **31–40 years**/*31-40 tahun* □ **41–50 years**/*41-50 tahun* □ **51 and over**/lebih daripada 51 tahun
**L3**	**Education Level:** *Tahap Pendidikan:* □ **SPM or SPTM or SPMV**/*SPM atau SPTM atau SPMV* □ **Diploma**/*Diploma* □ **Bachelor’s Degree**/*Ijazah Sarjana Muda* □ **Master’s Degree**/*Ijazah Sarjana* □ **Doctorate**/*Doktor Falsafah* □ **Others**/*Lain-lain*	**L4**	**Management Group** *Kumpulan perkhidmatan* □ **Top Management**/*Pengurusan Tertinggi* □ **Management and Professional**/Pengurusan dan Professional □ **Support Group**/*Kumpulan Sokongan*
**L5**	**Your experience using IoTbs.** *Pengalaman anda mengguna IoTbs.* □ **<5 years**/*<5 tahun* □ **5–14 years**/*5–14 tahun* □ **15–24 years**/*15–24 tahun* □ **>25 years**/*>25 tahun*	**L6**	**Department you are attached to.** *Jabatan yang sedang anda berkhidmat.* **----------------------------------------------------**

THANK YOU FOR YOUR PARTICIPATION/Terima kasih atas penyertaan anda.
